# Diagnostic Three Slides Pap Test Compared to Punch Biopsy and Endocervical Curettage in Confirmed HSIL+ Diagnosis

**DOI:** 10.3390/diagnostics11060942

**Published:** 2021-05-25

**Authors:** Roberta Rubeša-Mihaljević, Danijela Vrdoljak-Mozetič, Morana Dinter, Damjana Verša Ostojić, Snježana Štemberger-Papić, Marko Klarić

**Affiliations:** 1 Department of Pathology and Cytology, Clinical Hospital Center Rijeka, 51000 Rijeka, Croatia; danijela.vrdoljak.mozetic@kbc-rijeka.hr (D.V.-M.); morana.dinter@gmail.com (M.D.); dversa.ostojic@gmail.com (D.V.O.); snjezana.stemberger@ri.t-com.hr (S.Š.-P.); 2 Faculty of Medicine, University of Rijeka, 51000 Rijeka, Croatia; ginekologija@kbc-rijeka.hr; 3 Department of Obstetrics and Gynecology, Clinical Hospital Center Rijeka, 51000 Rijeka, Croatia

**Keywords:** diagnostic Pap test, punch biopsy, endocervical curettage, HSIL, final conus histology

## Abstract

Objective: The aim of the study was to evaluate the accuracy of the diagnostic Pap test (DPT) on three slides and punch biopsy and endocervical curettage (PB/ECC) compared with the final biopsy material in the detection of high-grade squamous intraepithelial lesion (HSIL). Materials and methods: Patients treated with conization after previous DPT and PB/ECC were analyzed. The findings of the DPT and PB/ECC as well as of the endocervical brush cytology and ECC were compared with the final conus histology. Results: 150 patients were analyzed, and final histology verified 145 cases of HSIL and 3 cancers. The percentage of confirmed HSIL cytology was 97%, while for PB/ECC it was 79% with 30/145 false negative results. The correlation between Pap test and PB/ECC showed that the diagnostic accuracy of DPT is significantly higher (*p* < 0.0001). Endocervical brush cytology confirmed HSIL+ in the endocervical canal in 83% and ECC in 35% of cases (*p* < 0.0001). Conclusion: The DPT on three slides enables better detection of HSIL compared to PB/ECC, particularly for lesions localized in the endocervical canal sampled with a cytobrush. A high quality DPT could represent a surrogate for PB/ECC and open the possibility of direct access to therapeutic procedure.

## 1. Introduction

Many countries are now moving toward high-risk human papilloma virus (HPV) screening as the primary test in cervical screening [1,2]. Therefore, there is a global tendency to replace the traditional Pap cytology screening of precancerous lesions and cancer with HPV testing due to its higher sensitivity and longer-term protection against high-grade cervical lesions [3,4]. In Croatia, cervical cancer screening has been present since the 1950s mainly as an opportunistic screening with estimated population coverage of 70%. It is performed using a Pap test predominantly as the primary screening test [5] and HPV as an adjunctive triage test. However, at the national level there is a future tendency to introduce HPV testing as a first-line primary screening tool, either simultaneously with cytology or as the only primary test in women aged 30 years or older [6,7].

The expanded use of HPV testing has given a new significance to cytology, which has become more accurate and specialized. It is well known that simultaneous cytological evaluation and HPV analysis significantly increases the efficiency of cervical cancer screening and reduces the overall incidence of invasive cancer [8]. In fact, due to high specificity, ability to identify abnormalities and correct prediction of the final histology outcome, the Pap test is now used as a secondary test, or triage test, after a positive HPV test [9,10]. Therefore, cervical cytology is currently even more implemented as a useful diagnostic tool that precedes colposcopy and histological confirmation [11].

According to Croatian national guidelines for the management of cervical intraepithelial lesions, colposcopy is recommended in patients with an abnormal Pap test detected in screening programs [12]. Afterwards, in women with colposcopically observed high-grade lesions, punch biopsy and endocervical curettage (PB/ECC) are suggested [12,13]. In the routine diagnostic practice at our institution, the approach to diagnosing cervical lesions includes repeating cytology at the time of colposcopy before doing PB/ECC. Therefore, a Pap test is performed as an additional diagnostic test (DPT). At that point, in order to ensure an optimal specimen that will accurately locate the lesion, cytological samples of the posterior fornix of vagina, ectocervix and endocervix are taken on three separate slides [14]. Furthermore, ancillary testing such as p16/Ki-67 dual staining and HPV testing may be performed in order to achieve the correct diagnosis. This gives the opportunity to the gynaecologist to have simultaneously a DPT and/or PB/ECC report as confirmation of high grade dysplasia before deciding the type of final excision treatment.

In order to assess the role of Pap tests in the management of cervical lesions, especially in the era of primary HPV screening, the aim of this study was to compare the results of DPT and targeted PB/ECC with the final histological diagnosis of squamous high-grade cervical lesions. In addition, the results of the endocervical brush cytology and endocervical curettage were correlated separately with the final histological finding. These results may contribute to determine better the position of cytology as a valuable diagnostic tool in the diagnostic management of cervical dysplasia.

## 2. Materials and Methods

### 2.1. Study Population

The study population was patients referred for colposcopy at the Clinic of Gynaecology and Obstetrics of the Clinical Hospital Centre Rijeka between January 2019 and December 2020 because of an abnormal Pap smear. Inclusion criteria for the study were repeated DPT taken at the time of colposcopy, PB/ECC at the time of colposcopy or in the next visit and subsequent cone biopsy treatment. Patients received large loop excision of the transformation zone (LLETZ) or cold knife cone biopsy treatment for final HSIL+.

### 2.2. Methods

Conventional DPT was taken at the time of colposcopy before histologic examination. The smear was placed on three separate slides representing the samples from the vagina, ectocervix and endocervix. Ayre ectocervical spatula and endocervical brush were used. The convex end of the Ayre spatula was used for the posterior vaginal fornix sampling with semicircular movement. The concave end was used for the ectocervix, and external orifice of the endocervical canal and sampling was done with complete circumference scraping. Endocervical brush cytology was performed by inserting a standard cytobrush to its full depth into the cervical canal. It was then rotated 90° to 180°. The material was transferred covering the whole glass slide. The slides were fixed in 95% ethanol and stained by routine Pap staining method. The resulting slides were reviewed and signed out by a cytopathologyst according to The Bethesda System for Reporting Cervical Cytology. HSIL cytological findings were classified positive, while no dysplastic changes or LSIL (low grade squamous intraepithelial lesion) were considered as negative for HSIL. In the group of ASC-H (atypical squamous cells—cannot exclude HSIL) additional testing with p16/Ki-67 dual-staining (CINtec^®^ PLUS Cytology, Roche Diagnostics, Basel, Switzerland) was performed, and the cases were included in the positive group. The location of the dysplastic cells in the specimen was determined semi-quantitatively (ranging from zero to three) to be in posterior fornix, ectocervical or endocervical.

Colposcopy was performed by a gynaecologist certified in colposcopy. Detailed colposcopic examination was performed after the application of 3% acetic acid. Colposcopic impression was classified as unsatisfactory; negative; abnormal, grade 1 (G1); abnormal, grade 2 (G2); and suspect for invasive cancer. Single or multiple colposcopically directed PB were taken from the worst areas of abnormality together with ECC. ECC was performed by placing the Kevorkian metal curette inside the endocervical canal. Gentle pressure was applied at its tip and the curette moved along the length of the endocervix while being rotated in circular way to sample the entire circumference of the canal. Specimens from the endocervical curettage were reviewed microscopically and the amount of endocervical material was described as scant, moderate or abundant.

Loop excision was performed following four-quadrant infiltration of local anaesthetic into the cervix. Specimens obtained by PB, ECC, LLETZ or cold knife cone procedure were immediately fixed in formalin and sent for histological examination. Histological examinations were performed by gynaecological pathologists following a standard protocol. Histological findings on cone specimens were used as the gold standard to measure the performance of the Pap test and colposcopically directed PB and ECC. In the case of HSIL+ diagnosis on PB and ECC with consecutive negative cone specimen, histology diagnosis made on PB and ECC was considered final. When multiple biopsies were taken, the highest-grade lesion was used for analysis. Similarly, if cone and LLETZ specimens showed different foci of varying grades of CIN, the worst grade was used as a final diagnosis. The location of the dysplastic process in the conization specimen was determined to be ectocervical or, if present within the endocervical canal, endocervical. Two patients reported as glandular atypia or with coexistence of both squamous and glandular pathology were excluded from the study since only squamous cell lesions were investigated.

### 2.3. Statistical Analysis

Categorical data was expressed as number and percentage. Chi-square (χ^2^) test was applied to compare proportions; *p* values < 0.05 were considered statistically significant. Analysis was done with the statistical software MedCalc (MedCalc Software, Version 20, Ostend, Belgium).

## 3. Results

For this study 150 patients met inclusion criteria and were analyzed. The patients’ ages ranged from 22 to 71 years with a median of 37 years (Table 1). A total of 127 patients were of reproductive age, and 23 were postmenopausal. Most patients with HSIL-detected lesions were in the age group between 35 and 39 years.

Cytology reports of DPT were evaluated, and the results are shown in Table 2. Most results of DPT were of high-grade dysplasia, including 116 cases of HSIL (77%), 29 of ASC-H (20%), 4 of LSIL (2.4%), 1 case of carcinoma and no ASCUS cytology report. In the group of ASC-H, ancillary testing with p16/Ki-67 dual staining was performed and resulted positive in 16 cases. The distribution of patients’ characteristics is shown in Table 2. Colposcopic assessments were recorded for 70 patients and detected mostly grade 1 (57%) and grade 2 lesions (22%). For colposcopic reports, 14% were classified as negative and 7% unsatisfactory, mostly due to the impossibility to visualize the squamocolumnar junction. Patients with abnormal Pap tests were assigned to PB and ECC, and the histologic results yielded 15 negative cases with no dysplasia (10%), 17 LSIL (11%), 118 HSIL (79%). Overall, 114 patients underwent LLETZ and 36 cold knife cone biopsy. Out of 150 patients, the final histologic findings confirmed 145 cases of HSIL and 3 cases of microinvasive carcinoma (Table 2).

Table 3 shows the results of the DPT compared with the final histological result of LLETZ or cold knife cone biopsy. HSIL cytological findings were classified positive, while no dysplastic changes or LSIL were considered as negative for HSIL. The overall percentage of positive Pap smears confirmed by final histology findings was 97% (142/146). There were only 4 false negative Pap smears confirmed as HSIL by final histology (3%).

Similarly to Pap tests, HSIL findings on PB/ECC were considered positive while no presence of dysplasia and LSIL classified as negative. As shown in Table 3 the agreement or percentage of positive PB/ECC confirmed by final histology was 79% (115/145). Both cytology as well as biopsy identified three cases of carcinoma. Interestingly, 30 punch biopsies resulted in false negatives since high-grade dysplasia was confirmed on final histologic diagnosis (20%).

When the diagnostic accuracy of DPT and PB/ECC was compared, a statistically significant difference was noted. DPT had a significantly higher prediction rate of HSIL lesions when compared to PB/ECC (*p* < 0.0001). Furthermore, biopsies taken at the time of colposcopy often yielded false-negative reports. In fact, the false-negative results were significantly higher among PB/ECC when compared to DPT, thus suggesting a better agreement between cytology and final histology in cone biopsy.

Next, we were interested in whether endocervical brushing might have better diagnostic value compared to ECC for identifying dysplastic lesions when the lesion is located in the endocervical canal. There were 92 cases with simultaneously performed ECC and endocervical brush that were analyzed and compared with the final histologic diagnosis. When the results of the endocervical brush findings were evaluated, 73 (83%) of the 88 patients had high-grade dysplasia in the endocervical sample confirmed on conization (Table 4). However, 17% of the endocervical cytology reports were interpreted as negative but confirmed as HSIL located in the endocervix on final histology.

**Figure 1:**
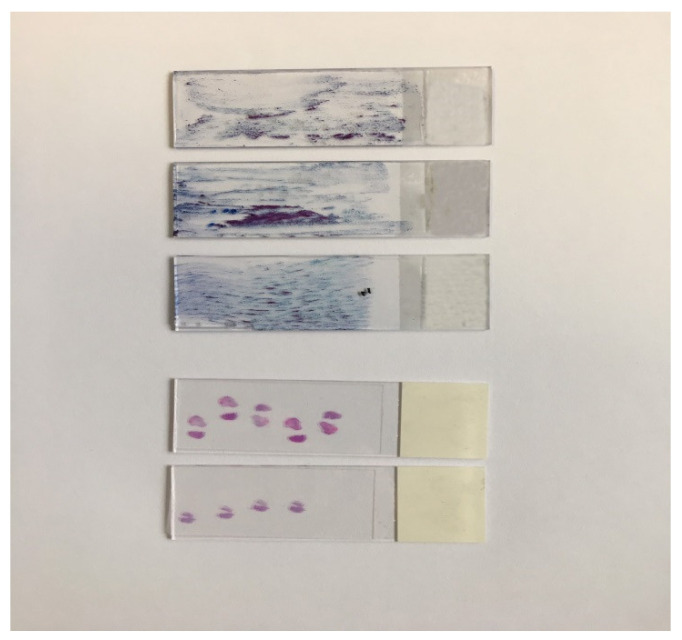
Diagnostic Pap test on three slides and histology tissue section of endocervical curettage material.

Disagreement between ECC and final histology was also evident. In fact, 65% of the ECC findings resulted in false negatives when compared to results of cone biopsy (Table 4). We were interested in whether false negative cases of ECC could be due to scant and inappropriate material. Therefore, ECC was analyzed in relation to the amount of endocervical material and quantitated as scant, moderate and abundant. The amount of material found on DPT and the histology tissue section of ECC can be observed in Figure 1. Indeed, 17% of all ECC samples were classified as scanty or borderline to ensure an accurate interpretation. All samples of endocervical brushed were satisfactory.

Although both DPT and ECC had a considerable level of false negative reports when compared, a significantly higher proportion of false negative results was noted in ECC (*p* < 0.0001). Therefore, the agreement between ECC and conization was only 35% since ECC detected only 29 HSIL cases out of 84 positive on conization (Table 4). Endocervical brush appeared to have better diagnostic accuracy since the proportion of true positive results was significantly higher when compared to ECC (*p* < 0.0001).

## 4. Discussion

In the era of primary HPV screening, cytology has achieved a new role and changed its position from a primary screening tool of a large population to a secondary specialized triage and diagnostic test inevitable in the algorithm of cervical intraepithelial lesions management [15,16]. In fact, one of the benefits of primary HPV testing is that HPV-negative patients are unlikely to develop precancerous lesions or cancer and are no longer expected to be referred to cytology [17]. On the other side, with primary HPV screening, a great increase in screen-positive results is expected. Therefore, methods for selecting HPV-positive women, who do not need an immediate colposcopy and have a low probability of carrying a colposcopy-detectable precancerous lesion, are necessary. A triage with cytology appears useful in order to avoid a large number of colposcopy referrals [18,19]. At this point, it is expected of cytology to dispose with highly educated and trained personnel as well as ancillary methods, such as immunocytochemistry protocols or cell blocks in order to correctly diagnose, triage and redirect HPV positive or negative patients [18].

The most frequent cause of misdiagnosis in cervical cancer screening is inadequate cervical sampling. The correct sampling of the cervix contributes significantly to the diagnostic value of the Pap test [20]. As previously suggested by other studies to assure an optimal cytological specimen [9,14], gynecologists at our institution have a long-standing practice of performing a DPT on three separate slides by taking the samples from the vagina, ectocervix and endocervix. In fact, our results confirmed that applying this technique offers a diagnostically excellent quality of the Pap test. When the results of the DPT were compared with the final histological result, the overall percentage of high-grade Pap smears confirmed by final histology findings was 97%, with only 4 false-negative results out of 145. To the contrary, the agreement between positive PB/ECC and conization was 79%. Other studies have already analyzed discrepancies between the Pap test, PB/ECC and final histology showing similar results. Ihonor et al. [21] confirmed that the agreement between PB and LLETZ was 61%, but according to the authors a significant correlation between PB findings and LLETZ still makes colposcopically directed PB a better predictor of CIN [21]. Other studies indicate that the Pap test is equally sensitive to histopathological examination [22,23]. In our study, DPT confirmed a significantly higher concordance rate of HSIL lesions when compared to PB/ECC.

There is a common perception that, because histology is the “gold standard” of diagnosis, non-correlating cytological results are always wrong. However, cytology offers certain advantages over histology in the assessment of cervical dysplasia. Applying the above-mentioned Pap test technique offers a diagnostically better quality sample by providing a greater amount of material and allowing the cytologist to correctly localize the lesion. In addition, previous authors claim that the morphology of intact cells in a cytological preparation is better to interpret subtle abnormalities in comparison to that of sectioned cells in histology that may not be visible in histologic preparations [22]. Finally, a good Pap test samples a broad area, while only a portion of the squamocolumnar junction may be sampled by biopsy [22].

When the results of the endocervical brush and ECC were evaluated, both DPT and ECC had a considerable level of false negative results, with 17% for endocervical brush and 65% for ECC. Previous studies have reported that ECC missed 45% of lesions in the endocervical canal identified on subsequent conization [24]. According to Chrysostomou et al., the false negative rate for ECC was 32.7% while for endocervical brush it was 8.4%. However, studies indicate that ECC has a higher degree of specificity compared to endocervical brush [17]. There is still no wide agreement on the importance of performing ECC. Factors supporting the necessity to apply ECC consider that ECC can detect lesions that otherwise might be missed by biopsy; ECC identifies lesions in the canal that can be more severe than the one on the portio and is needed for the recognition of a possible endocervical adenocarcinoma [24,25]. On the other side, the adequacy of the sample plays an important role in the detection of lesions in the canal. In fact, the inadequacy rate in ECC is high, with reports indicating 22%, mostly because of inefficient sampling and poor recovery of the histologic sections due to tissue processing and dilution [26]. In our study, 17% of all ECC samples were classified as scanty. Consequently, some authors support the idea that ECC is not indispensable and can be omitted in most instances and replaced by endocervical brush cytology [24,26]. Indeed, our results confirmed that the agreement between ECC and conization was only 35%, while endocervical brush appears to have better diagnostic accuracy since 83% of cytological HSIL lesions were confirmed in the conization specimen. Besides a higher adequacy rate and higher sensitivity for squamous lesions, recent research confirmed that endocervical brush cytology is more sensitive than ECC for detecting endocervical carcinoma, and with the additional use of a cell block and immunostaining it may be even more helpful [27]. Furthermore, endocervical brush has a higher adequacy rate compared to ECC, it is less expensive and it is associated with less patient discomfort [27].

The lack of concordance between cytology, colposcopically directed PB and subsequent histopathological conization finding is common and remains an important clinical problem. False-negative biopsy or biopsy results that underestimate the grade of CIN may have serious implications if a subsequent conization is not performed [23]. According to the consensus guidelines for the management of abnormal cervical cancer screening tests and cancer precursors released by the American Society for Colposcopy and Cervical Pathology for women with HSIL cytology immediate excisional procedure is acceptable [11] and some authors suggest a “see-and-treat” strategy even in women under age 25 [28]. The results of our study support a similar strategy since they indicate that cytology may offer certain advantages over histology in the assessment of cervical dysplasia. In fact, a well-sampled DPT allows the collection of cells from a broad area of the transformation zone and to localize correctly the lesion. Endocervical brush might replace ECC in evaluating the endocervical canal, particularly in cases when colposcopy is inadequate. Although it is important to remember that both cytology and PB/ECC are diagnostic procedures that are subject to variation in sampling, preparation and interpretation error, a high-quality HPV-informed cytology test offers better diagnostic performance and could possibly represent a surrogate for PB/ECC in the management of cervical dysplasia in the era of HPV primary testing.

## Figures and Tables

**Table 1:** Age distribution of patients enrolled.

Age (y)	Total Number of Patients	
	N	(%)
20–24	10	(6.6%)
25–29	22	(14.7%)
30–34	29	(19.3%)
35–39	40	(26.7%)
40–44	16	(10.7%)
45–49	10	(6.6%)
50–54	7	(4.7%)
55–59	7	(4.7%)
>60	9	(6%)

**Table 2:** Clinicopathological characteristics of patients.

Title	Title	Title
**Diagnostic Pap Test Findings**		
ASCUS	0	(0%)
LSIL	4	(2.4%)
ASC-H	29	(20%)
HSIL	116	(77%)
Carcinoma	1	(0.6%)
**Cervical Biopsy/Endocervical Curettage Findings**		
Negative	15	(10%)
LSIL	17	(11%)
HSIL	118	(79%)
Carcinoma	0	(0%)
**Colposcopic Findings**		
Negative	10	(14%)
Grade 1 (G1)	40	(57%)
Grade 2 (G2)	15	(22%)
Unsatisfactory	5	(7%)
**Final histologic Findings**		
Negative	1	(1%)
LSIL	1	(1%)
HSIL	145	(96%)
Carcinoma	3	(2%)

**Table 3:** Diagnostic Pap test and punch biopsy/endocervical curettage findings compared to final histologic diagnosis. (Abbreviations: DPT: diagnostic Pap test; PB/ECC: punch biopsy/endocervical curettage).

Final Histologic Diagnosis	DPT Findings *N (%)*		Total Number *N*	PB/ECC Findings *N (%)*		Total Number *N*
	Negative	Positive		Negative	Positive	
**Negative**	0	0	0	1	0	1
**LSIL**	0	1	1	1	0	1
**HSIL**	4 (3%)	142 (97%)	146	30 (20%)	115 (79%)	145
**Carcinoma**	0	3	3	0	3	3
Total	4	146	150	32	118	150

**Table 4:** Endocervical brush and endocervical curettage findings compared with final histologic diagnosis. (Abbreviations: ECC endocervical curettage).

Final Histologic Diagnosis	Endocervical Brush *N (%)*		Total Number *N*	ECC *N (%)*		Total Number *N*
	Negative	Positive		Negative	Positive	
**Negative**	0	1	1	2	1	3
**LSIL**	0	1	1	1	2	3
**HSIL**	15 (17%)	73 (83%)	88	55 (65%)	29 (35%)	84
**Carcinoma**	0	2	2	0	2	2
Total	15	77	92	58	34	92
